# Uncovering exposome-related diseases through the pathologic metallome: a novel approach for clinical populations

**DOI:** 10.3389/ftox.2025.1625874

**Published:** 2025-08-11

**Authors:** Riccardo Leinardi, François Huaux

**Affiliations:** Louvain Centre for Toxicology and Applied Pharmacology (LTAP), Institute of Experimental and Clinical Research (IREC), Université catholique de Louvain (UCLouvain), Brussels, Belgium

**Keywords:** metal toxicology, metallome, metallomics, metal exposure, exposome, chronic diseases, patients, clinical applications

## Abstract

Environmental exposure to complex metal mixtures plays a critical role in the onset and progression of diverse chronic diseases, in ways that the traditional toxicological framework fails to capture. A paradigm shift is underway, moving toward a more integrated understanding of combined metal effects through the interdisciplinary study of the metallome, the distribution of metal ions and metalloids within a biological system. In this perspective, we highlight the clinical importance of metallome to identify specific subpopulations in which disease onset or progression is primarily driven by environmental metal exposure rather than genetic predisposition. To achieve this goal, robust and sensitive analytical methods are required to overcome the limitations of conventional approaches and enable the detection of the full spectrum of metal species, including metals sequestered within mineral particles present in body fluids and tissues. We propose methodological innovations in sample preparation and analysis that expand the current scope of metallome-associated research. Together, these advances support a comprehensive framework for assessing metal mixture effects in environmental health, bridging toxicology with clinical practice and enabling more targeted, exposure-informed public health interventions.

## 1 Introduction

Over the past 15 years, epidemiological studies have highlighted the role of environmental stressors in influencing the development of human diseases, often acting as triggers of genetic predisposition (Munzel et al., 2021; Olden and Wilson, 2000). In 2005, the *exposome* concept was introduced by Christopher Wild and later expanded by Rappaport and Smith to encompass the totality of environmental exposures influencing human health (Wild, 2005; Rappaport and Smith, 2010; Dennis et al., 2017). Metals and metalloids - both naturally occurring and anthropogenic represent a significant component of the exposome due to their ability to bioaccumulate and exert toxic effects (Fu and Xi, 2020). While elements such as zinc (Zn), selenium (Se), molybdenum (Mo), and silicon (Si) are essential for physiological functions, they can become toxic at elevated concentrations (Vinceti et al., 2017; Plum et al., 2010; Vyskocil and Viau, 1999). Conversely, nonessential heavy metals like arsenic (As), cadmium (Cd), lead (Pb), and mercury (Hg) have no biological functions and are toxic even at low doses (Tchounwou et al., 2012). Exposure to these metals occurs not only in occupational settings (e.g., mining, metallurgy) but also through less recognized sources such as plastics, pesticides, tobacco smoke, and certain recreational activities (Ashraf, 2012; Gimeno-Garcia et al., 1996; Turner and Filella, 2021).

**FIGURE 1:**
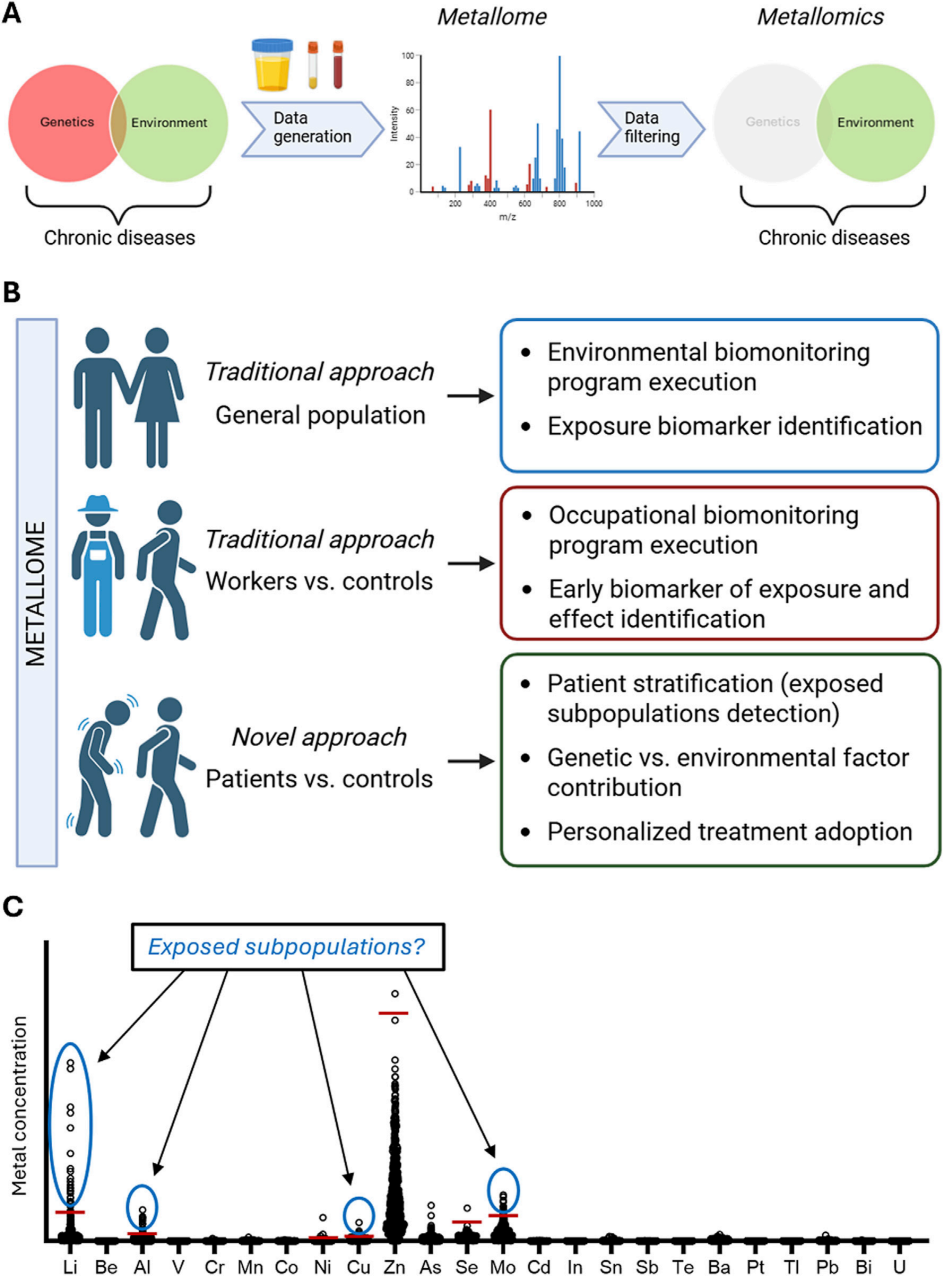
The metallome and metallomics as novel approaches to uncover the link between environmental exposure to metals and chronic diseases. **(A)** Chronic diseases are multifactorial conditions influenced by both genetic predisposition and environmental exposures throughout life, as well as their interactions. Characterizing the metallome in specific populations (e.g., healthy individuals, exposed workers, patients) through inductively coupled plasma tandem mass spectrometry (ICP-MS/MS) can help to reveal associations between metal exposure and the onset or progression of chronic diseases using metallomic approaches. **(B)** Overview of traditional and innovative applications of metallome analysis. In population-wide biomonitoring programs, metallome profiling supports exposure biomarker identification and reference value definition. In occupational settings, it allows early identification of exposure and effects. In clinical contexts, a novel approach compares patients and controls to detect exposed or susceptible subpopulations, supporting the investigation of gene-environment interactions and enabling personalized treatment strategies. **(C)** Example of inter-individual variability in trace metal concentrations across a patient cohort. Red horizontal lines indicate established upper reference limits from general population biomonitoring data. Several individuals show concentrations exceeding these limits (e.g., Li, Al, Cu, Mo), suggesting the presence of subpopulations with elevated exposure. This stratification enables the integration of metallome data with clinical phenotypes for patient-centered research. Red horizontal lines indicate established upper reference limits (URL) from general population biomonitoring data.

Toxicological research has been critical in characterizing the toxicity of metals and assessing their impact on human health. In this context, biomonitoring has emerged as a key tool to link environmental metal burdens with disease outcomes. In this perspective study, we propose the concept of the *metallome* – the dynamic network of metal and metalloid elements in the body – as a key feature in understanding metal-induced disease process (Haidar et al., 2023). Investigated within the framework of *metallomics* (Figure 1A), the metallome provides a system-level perspective to explore how metal mixtures influence biological pathways implicated in chronic diseases at the clinical level.

## 2 The historical approach in metal toxicology: one metal, one disease

The traditional “one metal–one disease” paradigm in metal toxicology has focused on studying individual metals and their specific pathological outcomes. This approach has provided key mechanistic insights and established links between metal exposure and disease. For instance, cadmium is a recognized nephrotoxicant associated with chronic kidney disease (CKD) - a progressive condition characterized by declining renal function (Byber et al., 2016; Kovesdy, 2011; Doccioli et al., 2024). Urinary and blood Cd levels consistently correlate with early kidney damage in both in the general and occupational populations (Roels et al., 1982; Roels et al., 1989; Roels et al., 1997; Bernard, 2004). Cobalt toxicity has been linked to cardiovascular dysfunction, likely through disruption of mitochondrial and myocardial energy metabolism and a dose-dependent association between Co exposure and cardiac effects is observed in exposed population (Lantin et al., 2013; Lodge et al., 2018). Manganese is recognized for its neurotoxic potential (Roels et al., 1987; Roels et al., 1992), and chronic Mn exposure in occupational contexts has been associated with neurobehavioral impairments (O'Neal and Zheng, 2015). These findings - establishing clear links between individual metals and specific pathologies - have been derived from a metal-by-metal investigative framework, in which each metal is examined independently. This metal-specific approach has also played a key role in establishing regulatory benchmarks, serving as a fundamental tool for defining environmental and occupational exposure limits. Two key metal-specific thresholds are commonly used in metal toxicology: reference values (RVs), indicating background metal levels in unexposed populations (Ewers et al., 1999), and biological limit values (BLVs), which define acceptable levels in occupational settings (Bolt and Thier, 2006). These thresholds are vital for interpreting biomonitoring data, identifying at-risk groups, and informing regulatory actions, playing a central role in preventing single metal-related health effects in both general and occupational settings (Angerer et al., 2007).

## 3 A new paradigm: combined mechanisms of multiple metals in low-exposure metal-associated diseases

The traditional single-metal toxicological framework does not adequately reflect the complexity of real-world exposure, where individuals are simultaneously exposed to multiple metals and other environmental stressors. This approach fails to capture potential interactions – synergistic, additive, or antagonistic – that may amplify or mitigate toxic effects. Recent epidemiological evidence suggests that many metal-associated pathologies result from combined exposures, even when individual metal levels remain within regulatory limits (Rodriguez-Barranco et al., 2013; Akinleye et al., 2024; Nordberg et al., 2022; Udom et al., 2025). Chronic low-level exposures, particularly common in industrialized and urban environments, can lead to bioaccumulation and long-term health risks, supporting the need for a paradigm shift toward mixture-based toxicology. Certain heavy metals such as Cd, Co, and Mn, target overlapping biological pathways, suggesting that co-exposure may disrupt shared metabolic or signaling networks (Haidar et al., 2023). For example, simultaneous co-exposure to low levels of Cd, Pb, and Hg has been associated with additive nephrotoxic effects (Akinleye et al., 2024), and several studies have documented increased risk of CKD and early signs of tubular damage in individuals concurrently exposed to both Cd and Pb, even when urinary concentrations remain below current safety thresholds (Cabral et al., 2015; Jain, 2019; Wang et al., 2024; Hambach et al., 2013). Occupational settings offer further evidence of mixture-specific toxicity. Lithium-ion battery (LIB) particles, primarily composed of lithium-cobalt oxides (LiCoO_2_) have been linked to systemic toxicity. Lithium is associated with nephrotoxic, thyrotoxic, and neuropsychiatric outcomes (McKnight et al., 2012), while cobalt contributes to cardiotoxicity (Brown et al., 2024). A recent biomonitoring study in LIB recycling workers found elevated urinary levels of both metals, and *in vivo* studies demonstrated that LIB particles induce greater pulmonary toxicity than that of either metal alone (Sironval et al., 2020; Hanser et al., 2022). Similarly, tungsten carbide–cobalt (WC–Co) mixtures, used in several industrial settings, are linked to hard metal lung disease, a fibrotic pneumoconiosis characterized by sustained oxidative stress, inflammation, and DNA damage (Lombaert et al., 2004; Lison et al., 1995; Nemery and Abraham, 2007; Norwood et al., 2001).

Several observational studies have explored the health effects of multi-metal exposure across diverse biological matrices (e.g., blood, serum, urine, seminal fluid). Many of these studies employ advanced statistical model, such as Bayesian Kernel Machine Regression (BKMR) and Weighted Quantile Sum (WQS) regression to characterize mixture interactions and associations various health outcomes. For instance, the NHANES cohort has been instrumental in exploring the relationship between metal mixtures and kidney, cardiovascular, and autoimmune outcomes (Wang et al., 2024; Guo et al., 2022; Feng et al., 2025; Chen et al., 2022; Liu H. et al., 2024).The MARHCS study in China examined how metals such as Li, Al, Fe, Zn, As, and Rb, influence semen quality in young men (Liu X. et al., 2024), while another large-scale study reported associations between heavy metal mixtures and impaired liver function (Zhao et al., 2022). Despite these advances, most studies are cross-sectional in design, which limit data interpretation as they cannot assess prior exposures or causality. This underscores the need for longitudinal and mechanistic approaches. Vulnerable populations – such as neonates, pregnant women, the elderly, and individuals with pre-existing conditions – also remain underrepresented, despite their potentially heightened susceptibility due to developmental or physiological factors. At the regulatory level, the EU agencies have issued guidance documents promoting tiered approaches to mixture risk assessment (de Jong et al., 2022; Galert and Hassold, 2021; More et al., 2019). These frameworks advocate moving beyond single-substance evaluations, to account for the additive or synergistic effects of co-exposures, consistent with the paradigm shift proposed in this perspective.

## 4 From general to clinical metallome: shifting toward patient-centered metal profiling

The metallome encompasses the entire repertoire of metal and metalloid ions, both free and protein-bound, within a cell, tissue, or organism (Zhou et al., 2024). This repertoire is dynamic and responsive, continuously shaped by factors such as environmental or occupational exposures, diet, lifestyle, and disease states. The definition and investigation of the metallome plays a central role in human biomonitoring and exposure science (Figure 1). In the general population, metallome profiling serves as a foundational tool for the execution of biomonitoring strategies (Hoet et al., 2013; Hoet et al., 2021). These efforts aim not only to assess exposure to environmental metals and establish reference values, but also to identify biomarkers that reflect often low-level, long-term exposure to complex environmental mixtures (Bocca and Battistini, 2024; Gil and Hernandez, 2015). Such applications provide valuable insights into background exposure trends and enable population-wide exposure assessments (Figure 1B).

In occupational settings, metallome analysis is typically applied to compare exposed workers with unexposed controls, supporting the identification of early biomarkers of both exposure and potential adverse effects (Zare Jeddi et al., 2021). Such strategies have advanced our understanding of how specific metal exposures relate to subclinical biological changes and early toxicological responses (Figure 1B).

Despite these advances, traditional biomonitoring approaches remain limited in fully elucidating the role of environmental metals in the onset and progression of chronic diseases. In this context, a novel and promising application of metallome analysis can emerge within the clinical domain, with a specific focus on patient populations. When altered in pathological contexts, the metallome becomes the *pathologic* metallome, offering insights into disease-specific metal dysregulation (Haidar et al., 2023). By comparing the pathologic metallome of patients with disease to that of healthy controls – and considering population reference levels for specific metals – this approach provides, in our view, a robust framework for unraveling the complex interplay between environmental metal exposure and genetic susceptibility into disease development, but also valuable opportunities for the advancement of personalized prevention and treatment strategies (Figure 1B). Furthermore, a key advantage of this multi-layered approach is the stratification of clinical cohorts into subgroups with distinct exposure profiles, potentially linked to differential disease risk, trajectories, or therapeutic responses. Figure 1C illustrates this concepts: individual concentrations of 24 trace metals in urine are shown for a clinical cohort, with red lines indicating upper reference limits from a general population biomonitoring study (Hoet et al., 2013). For several elements (i.e., Li, Al, Cu, and Mo), distinct subgroups of individuals exceed these thresholds, suggesting the presence of exposed subpopulations not visible through average-based analysis. This scenario underscores the potential of metallome profiling in revealing hidden variability within clinical cohorts, enabling the identification of individuals with atypical exposure burdens. Integrating this stratification with clinical phenotypes enhances the ability to define environmental and genetic contributions to disease, offering novel insights into pathogenesis and paving the way for targeted therapeutic strategies.

**FIGURE 2:**
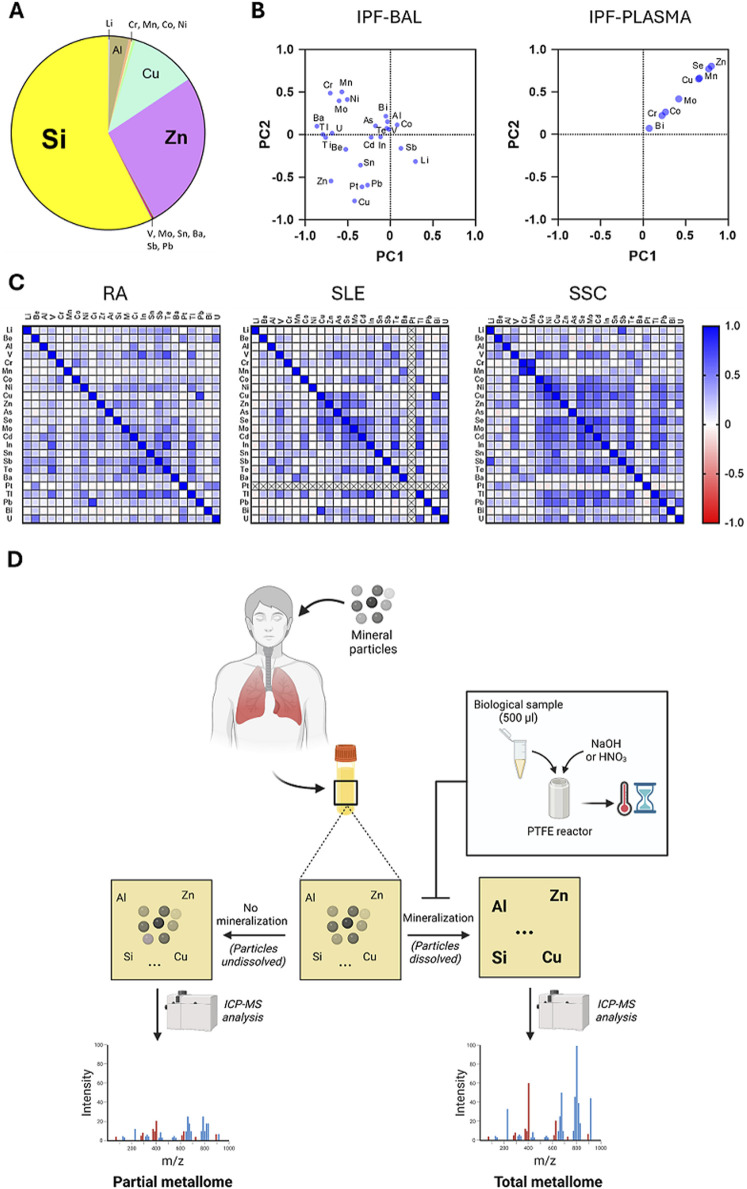
Distinct associations of metals in body fluids from patients with chronic diseases associated with environmental factors. **(A)** ICP-MS/MS quantification of metals in biological samples (bronchoalveolar lavage–BAL) from patients with idiopathic pulmonary fibrosis (IPF) reveals a predominance of silicon (Si) relative to other tracers. **(B)** Principal component analysis (PCA) of BAL (left) and plasma (right) samples from IPF patients identifies distinct metal crulters, supporting a potential role for specific metal associations in disease pathogenesis and/or progression. **(C)** Patients with rheumatoid arthritis (RA), systemic lupus erythematosus (SLE), and systemic sclerosis (SSc), exhibit disease-specific urinary metallome profiles. Pearson correlation analysis highlights unique metal-metal association patterns across the three conditions, suggesting that specific metal clusters may play a role in the pathogenesis or progression of each autoimmune disease. **(D)** Alkaline (NaOH) and acidic (HNO_3_) mineralization strategies may assist in detailing the metallome in exposed populations considering the contribution of metal-containing particles of environmental origin accumulated into body fluids (urine, blood, plasma) following pulmonary deposition and biodistribution in extrapulmonary targets.

More specifically, the pathologic metallome profiling on bronchoalveolar lavage (BAL) and plasma samples (Figure 2A) from idiopathic pulmonary fibrosis (IPF) patients indicated silicon (Si) as the predominant element in BAL fluid, consistent with documented occupational exposure to silica-containing dust, a known IPF risk factor (41, 42). Additionally, about 20% of patients had elevated plasma levels of copper (Cu) and zinc (Zn). Copper dysregulation has been proposed to play a mechanistic role in fibrotic lung diseases, acting both as an exposure biomarker and contributor to pathogenesis (43). Principal Component Analysis (PCA) of elemental profiles (Figure 2B) revealed distinct metal associations in BAL and plasma samples, supporting the idea that multiple metals may act synergistically or additively in IPF onset or progression. Similarly, as part of the H2020 project “EXIMIOUS”, we profiled urinary metallomes in patients with rheumatoid arthritis (RA), systemic lupus erythematosus (SLE), and systemic sclerosis (SSc), three autoimmune diseases believed to arise from complex interactions between environmental and genetic factors (Costenbader et al., 2012; Choi et al., 2024). Pearson correlation analyses revealed distinct metal association patterns unique to each condition (for instance, Pb-Cu and Cr-Co for RA, Te-In and Zn-Se for SLE, Li-Sb and Cr-Mn for SSc), suggesting that specific metal clusters may influence disease pathogenesis or progression (Figure 2C). To further clarify these relationships, future studies integrating metallomic data with bio-clinical parameters such as cytokine levels, immune cell phenotypes, and epigenetic modifications will be essential. Metallome profiling can uncover disease endotypes linked to specific metal exposures that remain hidden in genetically or clinically heterogeneous populations. Building on this, we propose a conceptual framework for incorporating metallome profiling into research on non-communicable diseases (NCDs). By capturing exposure-driven pathophysiological signatures at diagnosis, this method could help identify patients whose disease onset or progression is influenced by environmental factors, enabling more personalized prevention and treatment strategies, from targeted exposure mitigation to customized therapies. Altogether, this approach positions the metallome as a key tool at the intersection of exposure science, toxicology, and precision medicine.

## 5 Analytical approaches to measuring the metallome

In biological fluids, trace metal metallome is typically measured using established analytical methods such as atomic emission spectroscopy (AES), anodic stripping voltammetry, X-rays techniques, nuclear methods, and mass spectrometry, with inductively coupled plasma mass spectrometry (ICP-MS) and optical emission spectroscopy (ICP-OES) being among the most widely applied (Zhou et al., 2022; Brown and Milton, 2005). The importance of these techniques in clinical scenarios, where accurate detection of metals at trace and ultra-trace levels – typically in the parts-per-billion (ppb) range – is essential. ICP-MS, which offers the possibility to simultaneously quantify multiple different metals within a single biological matrix, has demonstrated robust analytical performance over the years. It has been successfully employed in the analysis of a broad range of biological specimens (Zhou et al., 2024). In ICP-MS, the analysis of biological samples often requires a prior mineralization step, a chemical digestion process typically involving strong acids to break down complex organic matrices. This step is essential to convert biological tissues or fluids into a clear solution, thereby reducing matrix effects, minimizing the risk of clogging or damage to instrument components, and ultimately enhancing analysical sensitivity, accuracy, and reliability. Microwave-assisted mineralization methods have been developed to facilitate the definition of the metallome in human samples including blood and tissues (Grassin-Delyle et al., 2019). However, mineralization protocols should be designed and optimized not only for organic matter digestion, but also to ensure the dissolution of environmental particles (i.e., silica) which may be present in biological matrices, converting them into soluble, measurable forms. These particles, potentially introduced into the body to various exposure routes and subsequently biodistributed across tissues depending on their physico-chemical characteristics (Leinardi et al., 2025), can carry metals that contribute to the overall individual metal burden. By adopting this comprehensive approach, it becomes possible to define a more inclusive or “total” metallome, which encompasses both solubilized metals species in biological samples, and particle-associated metals derived from prior exposures, thereby providing a more accurate representation of an individual’s total metal exposure (Figure 2D).

Hydrofluoric acid (HF) is commonly used for this purpose, due to its capacity to dissolve mineral particles. However, HF presents several drawbacks, including technical complexity and the formation of interferent species (Bossert et al., 2019; Arslan and Lowers, 2024). To address these limitations, we have recently developed two novel mineralization protocols the represent an important methodological advancement: an alkaline digestion using sodium hydroxide (NaOH), and an acidic digestion with nitric acid (HNO_3_) (Figure 2D). These alternative methodologies have been successfully applied to blood and urine samples from various subject groups, including patients, workers, control individuals and in the general population. Both methods ensure effective mineralization of biological matrices and the dissolution of ultrafine mineral particles, including silica and various metals, found in body fluids or tissues (Leinardi et al., 2025). These innovative protocols provide valuable new tools for biomonitoring the metallome in populations exposed to environmental mineral dust (Figure 2D). Furthermore, combining these mineralization approaches with routine ICP-MS analysis enables the detection, quantification, and characterization of individual metallic particles dispersed in biological samples. This integrated approach would offer new opportunities to investigate the potential role of particulate metals in modulating the metallome, and their contribution to exposure-related health outcomes in affected subjects.

## 6 Metallomics and omics synergies in the study of metal mixture effects on human disease pathways

The emerging fields of metallomics and the metallome represent powerful frameworks for understanding the complex biological effects of metal exposures, particularly in the context of environmental co-exposures. These disciplines support a shift away from traditional single-metal toxicology, enabling the investigation of multiple metals simultaneously, along with their speciation, distribution, and interactions within biological systems. Introduced in the early 2000s by Haraguchi as part of the broader “-omics” paradigm, metallomics has evolved into an interdisciplinary approach focused on the comprehensive, quantitative, and functional analysis of metals and metalloids in biological systems. Recent advances in high-throughput analytical technologies and computational biology have enabled the integration of metallomics with other omics disciplines, including genomics, transcriptomics, proteomics, metabolomics, ionomics, and speciomics (Haraguchi, 2017). This multi-omics integration allows for the elucidation of metal-induced molecular mechanisms that contribute to disease pathogenesis, particularly in scenarios involving metal mixtures. While traditional toxicokinetic models often fail to capture these interactions, metallomics provide insights into metal speciation and compartmentalization, while complementary omics techniques reveal downstream gene, protein, or metabolite perturbations. The coupling of speciation-based metallomics with transcriptomic profiling enables researchers to identify the biologically active forms of metals responsible for toxicity, and link them to specific cellular effects. Gene and protein expression analyses can detect early molecular signs of toxicity (Korashy et al., 2017), while machine learning and multivariate modeling can uncover metal-associated patterns, co-exposure profiles, or predictive molecular fingerprints.

Integration with other omics disciplines has already yielded valuable insights. Metallomics-metabolomics studies have revealed key mechanisms by which metals contribute to metabolic disorders (Akash et al., 2023). Metal exposures have also been shown to influence endogenous microRNA activity (Wallace et al., 2020) and interact with genetic polymorphism in metal transporter genes (i.e., MT1 and SLC30A), affecting individual susceptibility to metal toxicity (Lai et al., 2024). The complex relationship between metal profiles and cancer has been investigated through the integration of metallomics/ionomics with transcriptomics, proteomics and machine learning approaches, suggesting the possibility that alterations in some elements (i.e., Zn, Mn, and Cu) can influence key biological processes ultimately leading to carcinogenesis (Callejon-Leblic et al., 2019). Beyond mechanistic insight, these integrative approaches support biomarker discovery, especially for identifying unique molecular signatures associated with binary or ternary metal co-exposures. Such signatures can be used to develop predictive models that distinguish between different exposure profiles (Wu and Xie, 2025). Machine learning and tailored data integration strategies further enable the generation of exposure-specific biomarker panels, improving risk assessment and informing public health interventions (Zare Jeddi et al., 2022). These biomarkers offer not only early detection of toxicity, but also guidance for targeted interventions in vulnerable or exposed populations.

## 7 Conclusion

This perspective paper highlights the evolving understanding of metal exposure as a significant concern in toxicology and public health, emphasizing the necessity to move towards an innovative paradigm which highlights the concept of multiple metal co-exposure in contributing to the development of a set of chronic pathologies in humans. Metallomics and metallome research represent precious tools for addressing the complexity of real-world metal exposures and provide valuable insights into how environmental exposure influence the pathogenesis of a multitude of non-communicable diseases. The active combination of metallomics with modern omics technologies may facilitate in addressing the significant challenges characterizing the modern *exposome-based* toxicology, including the impact of combined metal stressors, and the development of metal-specific quantitative adverse outcome pathways, providing more comprehension of the relationships between initiating and key events, and the resulting outcomes. In distinct metal-exposed populations, the integration of analytical techniques into biomonitoring and clinical investigations may enable the identification of previously non-detectable exposure risks, supporting the development of personalized assessment strategies, targeted disease prevention efforts, and precision therapeutic interventions.

## Data Availability

The raw data supporting the conclusions of this article will be made available by the authors, without undue reservation.
